# Biological forms of *Anopheles stephensi* in the city of Djibouti, Horn of Africa

**DOI:** 10.1371/journal.pone.0355973

**Published:** 2026-08-14

**Authors:** Gabriel Haddad, Osman Abdillahi Guedi, Nicolas Gomez, Sébastien Briolant

**Affiliations:** 1 American University of the Middle East, Egaila, Kuwait; 2 Centre de Recherche en Sciences Humaines, Sociales, Langues et Littérature, Université de Djibouti, Campus de Balbala, Croisement RN2-RN5, Djibouti, Djibouti; 3 Laboratoire de recherches Océan Indien: Espaces et Sociétés (OIES), Université de La Réunion, Saint-Denis, France; 4 Unité Parasitologie et Entomologie, Département Risques Vectoriels, Institut de Recherche Biomédicale des Armées, Marseille, France; 5 Unité Mixte de Recherche Risques Infectieux Tropicaux et Microorganismes Emergents, Assistance Publique-Hôpitaux de Marseille, Service de santé des armées, Université Aix Marseille, Marseille, France; 6 Institut Hospitalo-Universitaire Méditerranée Infection, Marseille, France; Quinnipiac University, UNITED STATES OF AMERICA

## Abstract

**Background:**

*Anopheles stephensi* is an invasive malaria vector found in South Asia and the Middle East. It was first described in Djibouti in 2012. It has since been linked to an increase in malaria cases and its spread to several African countries in the region. Given that the three biological forms of *An. stephensi* – Type, Intermediate, and Mysorensis – have different vectorial capacities and habitats, it is essential to know which forms are present in urban areas in Africa and which are responsible for malaria transmission.

**Methods:**

In April 2023, in the city of Djibouti, a total of 600 eggs of *An. stephensi* were collected from six sites and analyzed using a scanning electron microscope to identify the biological forms based on the number of ridges. Sequence polymorphism in the intron I region of the odor-binding protein 1 (*Obp1*) gene was also analyzed in 153 specimens of *An. stephensi*. The distribution of biological forms of *An. stephensi* across the six sites was also analyzed in light of the incidence rate of malaria in the corresponding areas.

**Results:**

At all sites, the number of ridges observed on the eggs corresponds with all three biological forms of *An. stephensi* living sympatrically in Djibouti. In peri-urban and urban areas, even in areas with high malaria incidence, the majority of eggs had ridge counts consistent with the Mysorensis form, which is described as being associated with rural areas and low vector capacity. In addition, there was no correlation between morphological analysis and molecular analysis of Obp1.

**Conclusions:**

All biological forms of *An. stephensi* are present in the city of Djibouti, where their role in malaria transmission requires further study in order to improve malaria control policies. Furthermore, *Obp1* is not a suitable molecular tool for distinguishing the three biological forms of *An. stephensi*.

## Introduction

Despite decades of effort, malaria remains a major public health challenge in 83 countries around the world, with most deaths occurring in Africa. According to the World Malaria Report, there were 263 million cases of malaria and 597,000 malaria-related deaths worldwide in 2023 [1]. Historically, malaria was considered a disease that only affected rural areas. Due to ongoing immigration and development, cities now have large areas of urban agriculture and are experiencing uncontrolled urban sprawl with poor water management, which provides a breeding ground for *Anopheles* species [2], including *Anopheles stephensi* [3].

Until 2010, *An. stephensi* was mainly described as a malaria vector in South Asia and certain neighboring countries on the Gulf Peninsula [4]. In 2012, it was detected for the first time in Djibouti [5], a country located in the Horn of Africa where it is responsible for *Plasmodium falciparum* and *P. vivax* transmission in humans [6]. Since then, its presence and rapid spread have been reported in several African countries [7–11] and have been clearly linked to the spread of malaria in the region [12,13].

Three biological forms of *An. stephensi* have been described: the Mysorensis, Intermediate and Type forms [14,15]. Many studies have sought to establish a correlation between these forms and their survival in rural or urban areas, as well as their ability to transmit *Plasmodium* parasites [14,16]. They associated the Mysorensis form with rural areas and its low vector capacity [15], while the Type and Intermediate forms were detected in both urban and rural areas, with a higher vector capacity [14,16,17].

In the 1970s, cases of malaria appeared in Djibouti along roads coming from neighboring countries, with *An. nili* being the main vector [18], then in urban areas, particularly in the district of Ambouli. During this period, malaria became endemic, with annual transmission in urban areas [19], where *An. arabiensis* was considered the main vector [18]. Since the 1980s, the country has actively launched campaigns aimed at reducing the number of malaria cases [20,21]. Despite a few intermittent outbreaks throughout the 1990s, the number of patients gradually declined, reaching 24 cases in 2012, when the country was in the pre-elimination phase of malaria. However, it increased in Djibouti in 2013, coinciding with the arrival of *An. stephensi* [5].

The main objective of this study was to identify the different biological forms of *An. stephensi* in the city of Djibouti by determining the number of egg ridges. The second objective was to analyze their correlation with habitat type and polymorphism in the intron I region of the odor-binding protein 1 (*Obp1*) gene.

## Methodology

### Study area and eggs collection

**Fig 1:**
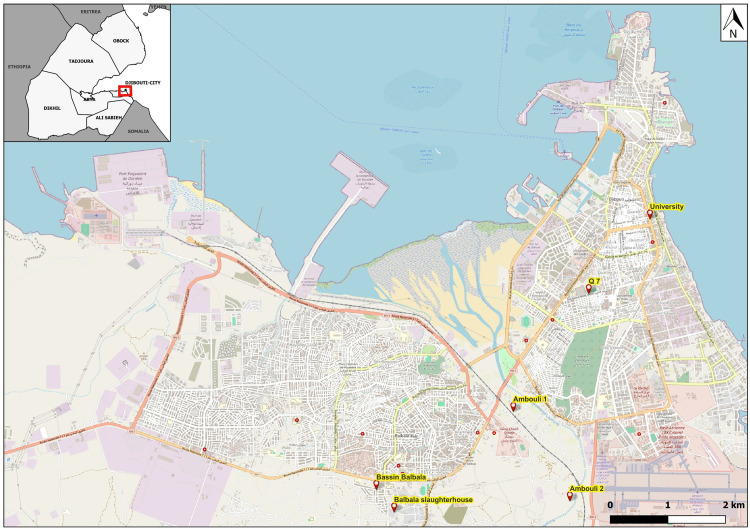
Map of Djibouti city showing the locations where eggs were collected. Base map: OpenStreetMap contributors, available under the Open Database License (https://www.openstreetmap.org/copyright).

The study was conducted in April 2023, in the city of Djibouti, capital of the Republic of Djibouti, in the Horn of Africa, where average daytime temperatures range from 17 °C to 42 °C and relative humidity ranges from 40% to 90%. A total of 600 *An. stephensi* eggs were collected from six water points, by immersion using a 350 ml ladle (for each sample, 500–1000 ml of water were transferred into vials and transported to the laboratory). Three of them are located in urban areas: Q7 (11.579944 N, 43,141879 E) and the University of Djibouti (11.581824 N, 43.151619 E) are located in the Boulaos district, and the Balbala basin (11.551887 N, 43,101472 E) is located in the Balbala district. The other three are located in semi-urban areas: Ambouli 1 (11.561178 N, 43.129878 E) and Ambouli 2 (11.546612 N, 43.139127 E) are located in the Ambouli district, and the Balbala slaughterhouse (11.545173 N, 43.110923 E) is located in the southern part of the Balbala district (Fig 1). From the water samples, four hundred and forty-seven eggs from the six sites were used for more detailed morphological analyses to assess the presence of different biological forms using a scanning electron microscope (SEM). For each of the six water collection, the mosquito larvae were hatched in mineral water. The larvae were then raised on a standardized daily diet of fish food in 24 × 34 × 9 cm plastic tanks and kept at ambient temperature and humidity (28°C, 75% relative humidity) until the adults emerged. All *Anopheles* adult female mosquitoes of the F0 generation were identified as *An. stephensi* on the basis of a morphological identification key [22]. One hundred and fifty-three adults *An. stephensi* of the F0 generation from the water collection of Ambouli 1 were frozen until further molecular analysis.

### Ethical statement

This study on *An. stephensi* eggs did not require ethical approval, as it involved no human interaction or animal intervention, in accordance with current regulations. The collection of eggs and larvae in domestic settings was carried out with the consent of the owners.

### Egg float ridges count using scanning electron microscopy

Standard microscope glass slides were covered with double-sided carbon adhesive tape with an aluminum base (Nisshin EM Co, Tokyo, Japan). *Anopheles stephensi* eggs collected were transferred to the tape and fully air-dried before SEM imaging. Micrograph were acquired on a TM4000Plus II Tabletop SEM (Hitachi High-Tech, Japan) at 150X magnification, using the backscattered electron detector with an accelerating voltage of 10kV, at a working distance of between 4.9- and 6.5-mm.

**Fig 2:**
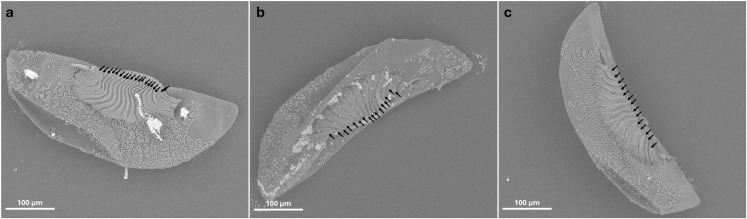
Scanning electron micrograph of lateral aspect showing floats and ridges of (a) *Anopheles stephensi* ‘type’ or A form egg, (b) *Anopheles stephensi* ‘intermediate’ or B form egg, and (c) *Anopheles stephensi* ‘mysorensis’ or C form egg. Micrograph were acquired at a 5 mm working distance, using BSE detector and a 10kV electron beam. Scale bar: 100 µm.

After micrograph acquisition, the number of float ridges was manually counted on each egg(Fig 2). Biological forms were determined according to the number of ridges as follows: 10–14 ridges for ‘mysorensis’ or type C form egg, 15–16 ridges for ‘intermediate’ or type B form egg, and 17–22 ridges for the ‘type’ or type A form egg as previously described [23,24]. Eggs with broken ridges or missing floats were excluded from the analysis.

### Sequencing of the Obp1 and COI genes

One hundred and fifty-three adults *An. stephensi* of the F0 generation from the water collection of Ambouli 1 were crushed in a TissueLyser II (Qiagen S.A.S., Courtaboeuf, France) and DNA was extracted as previously described [25]. To confirm the morphological identification of *An. stephensi*, sequencing of the cytochrome oxidase I gene was performed as previously described in de Santi et al. [6]. The primers and PCR parameters used to amplify a 845 bp fragment of the odorant binding protein 1 gene (Obp1) were similar to those described in Khan et al. [26]. Purification of PCR products and sequencing were outsourced to Microsynth (Vaulx-en-Velin, France). Each fragment was sequenced from both 5′ and 3′ ends. A 120 bp fragment from the Obp1 intron I region obtained from samples sequenced was used for analysis. The sequences of the *An. stephensi* sibling species A (KJ557463), B (KJ557452), C (KJ557455) were used as representatives for sequence comparisons with Geneious Prime v2022.2.2 software.

### Malaria incidence rate in the city of Djibouti in 2022

**Fig 3:**
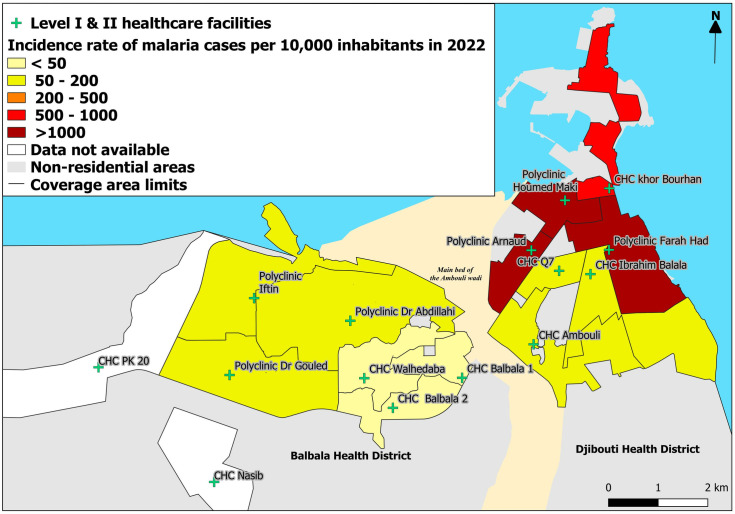
Map of Djibouti city showing the incidence rate of malaria cases per 10,000 inhabitants in 2022.

In Djibouti, the lack of a reliable addressing system (streets, house numbers) makes it difficult to accurately identify the place of residence of feverish patients consulting health facilities. To overcome this difficulty, the neighborhoods surrounding level I and II health facilities were grouped into service areas, corresponding to the area covered by each health center. Based on data from the 2022 health statistics directory [27], the malaria incidence rate in each radiation zone was then calculated per 10,000 inhabitants per year (Table 1). Malaria incidence data were mapped at the level of the 15 health center coverage areas using QGIS software version 3.44.4 and the Jenks discretization method (Fig 3).

**Table 1:** Malaria incidence rate per 10,000 inhabitants in 2022 in the areas covered by each health center in the city of Djibouti.

Coverage area	Population	Malaria cases	Incidence rate (/10,000 inhabitants/year)
CHC Walhedaba	38850	104	27
**CHC Balbala 2**	65487	50	8
Polyclinic Dr. Abdillahi	82372	429	52
**CHC Q7**	43388	373	86
Polyclinic Iftin	24279	148	61
Polyclinic Houmed Maki	38333	5127	1337
Polyclinic Farah Had	57859	9466	1636
Polyclinic Dr. Gouled	43910	790	180
**Polyclinic Arnaud**	36310	5651	1574
**CHC Balbala 1**	71198	37	5
CHC Ibrahim Balala	26329	460	175
**CHC Khor Bourhan**	25649	1468	572
**CHC Ambouli**	34658	507	146
CHC Nasib	NA	NA	NA
CHC PK 20	NA	NA	NA

CHC = Community Health Centre; NA: Not available.

## Results

**Fig 4:**
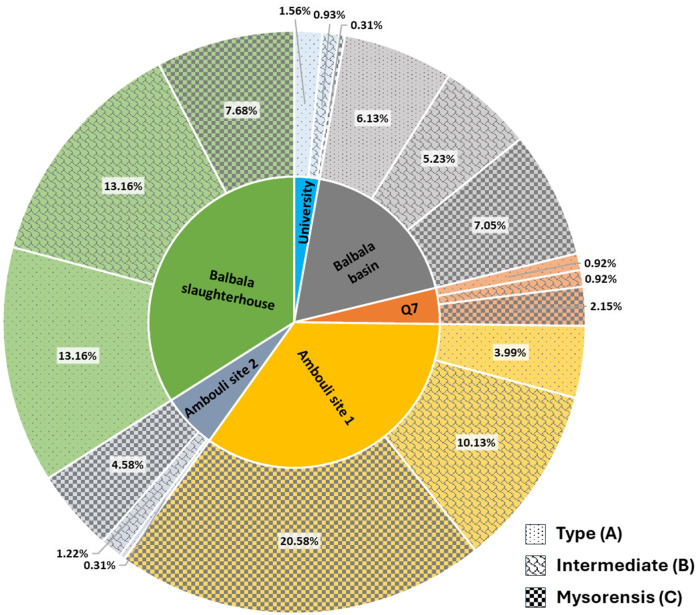
Distribution of the three biological forms of *Anopheles stephensi* in the six analyzed sites. Percentages shown are the proportions from the total number of *Anopheles stephensi* eggs analyzed using SEM.

A total of 600 *An. stephensi* eggs were collected from the six sites. The majority of these were collected in the districts of Ambouli (335/600, 55.8%) and Balbala (239/600, 39.83%) (Fig 4). Four hundred and forty-seven eggs from the six sites were analyzed using a scanning electron microscope (S1 File). The biological form could be determined for 326 eggs based on the number of ridges present on the floaters (Fig 2).

In the Ambouli 1 site, the majority of the eggs analyzed were categorized as Mysorensis (C) form (67/113, 59.3%) followed by the intermediate (B) form (33/113, 29.2%) and a minority belonged to the Type (A) form (13/113, 11.5%) (Table 2, Fig 4). A similar distribution was observed in Ambouli 2 collection site, with 15 specimens (15/20, 75%) classified as C form, 4 specimens (4/20, 20%) as B form and 1 specimen (1/20, 5%) as A form. At the Q7 collection site, the distribution was 7 specimens (7/13, 53.8%) for type C, 3 specimens (3/13, 23.1%) for type B, and 3 specimens (3/13, 23.1%) for type A. The Balbala basin showed almost similar proportions between form A (20/60, 33.3%), form B (17/60, 28.4%) and form C (23/60, 38.3%). In the Balbala slaughterhouse, the type form (or A) and the intermediate form (or B) were equivalent (43/111, 38.7%), while the proportion of the mysorensis form (or C) was lower (25/111, 22.6%). Finally, at university, form A was the most common with 5 specimens (5/9; 55.6%), while forms B and C accounted for 3 specimens (3/9, 33.3%) and 1 specimen (1/9, 11.1%) respectively (Table 2, Fig 4).

**Table 2:** Proportions of the three biological forms of *Anopheles stephensi* in the water collections for each of the different sites.

		Biological form of *An. stephensi* by SEM			
Sites		Type (A)	Intermediate (B)	Mysorensis (C)	Total
Urban	University	5 (55.6%)	3 (33.3%)	1 (11.1%)	**9 (2.8%)**
	Q7	3 (23.1%)	3 (23.1%)	7 (53.8%)	**13 (4%)**
	Balbala basin	20 (33.3%)	17 (28.4%)	23 (38.3%)	**60 (18.4%)**
Semi-urban	Ambouli site 1	13 (11.5%)	33 (29.2%)	67 (59.3%)	**113 (34.7%)**
	Ambouli site 2	1 (5%)	4 (20%)	15 (75%)	**20 (6.1%)**
	Balbala slaughterhouse	43 (38.7%)	43 (38.7%)	25 (22.6%)	**111 (34%)**
**Total**		**85 (26.1%)**	**103 (31.6%)**	**138 (42.3%)**	**326 (100%)**

Of the 153 *An. stephensi* sequenced for the COI gene, all were confirmed as *An. stephensi*. The *Obp1* gene was successfully sequenced for 143 adults *An. stephensi* from site 1 in Ambouli. It showed a majority of the intermediate form at 58.7% (84/143), followed by the type form at 40.6% (58/143). The mysorensis form was detected in one of the eggs analyzed, representing 0.7%.

## Discussion

The morphological analysis of *An. stephensi* eggs in this study yielded very different results depending on the sites analyzed. The six sites showed coexistence of the three biological forms in different proportions, which is consistent with what has been previously described in the literature [15,28].

With regard to peri-urban areas, sites 1 and 2 in Ambouli revealed a large majority of identified mysorensis forms, which is consistent with what has been described previously [14,15]. However, in the Balbala slaughterhouse, this study showed contradictory results compared to the conventional idea of the urbanization trend of the *An. stephensi* “Type” form. The validity of this concept can therefore be questioned in light of these results. In fact, previous research had already cast doubt on the accuracy of this hypothesis. As described by Oshaghi et al., only the mysorensis form has been found in urban and rural areas and in different climates in the regions most affected by malaria in Iran [28].

Moving toward more urban areas, the university presented a majority of “Type” forms, previously associated with malaria transmission and urban areas [2,15]. However, at site Q7, the majority of eggs analyzed were surprisingly classified as mysorensis, the biological form generally associated with rural areas and low transmission capacity [15]. Similarly, the Balbala basin showed an almost equal distribution of the three forms, with a slightly higher incidence of the mysorensis form. A significant number of malaria cases have been reported in these areas, mainly at the university and in the Q7 neighborhood, according to epidemiological data from the Ministry of Health and the results of a study conducted by Moussa et al. [27,29]. Thus, the results presented here reinforce skepticism about the association of the mysorensis form with rural areas and its low transmission capacity. In fact, scientists had been skeptical of this idea since it was first introduced by Sweet and Rao [14]. According to an experimental study conducted in 1939, there is no discernible difference between the susceptibility to infection of the mysorensis form and that of the Type form [16]. The paradigm shifted again in favor of the low infectious capacity of mysorensis form with Subbarao’s detailed description [15]. More recently, several studies based on morphometric methods have identified mysorensis form only in malaria-endemic regions of Iran [28], and Pakistan [26].

On the other hand, given that these three biological forms show similarities throughout their life cycle, except for the morphology of their eggs (more specifically the number of ridges), some research groups have attempted to identify factors associated with the number of ridges. Subbarao et al. demonstrated [15]: a) that the number of ridges on egg-float appears to be associated with ecological variation (a gradient of increasing ridges on egg-float is observed as one moves from rural to urban habitats), b) that this phenomenon is likely multigenic, and c) that the different biological forms of *An. stephensi* do not differ in terms of chromosome inversion frequencies, as also reported by Suguna [30]. Suguna et al. also demonstrated that the number of ridges on egg-float may be inversely proportional to the salinity of the breeding site of the species *An. subpictus* within the complex [31]. Several other groups of researchers also studied the possibility of identifying genetic differences. The sequence of intron I of *Obp1* in *An. stephensi* has been identified as a suitable molecular marker for distinguishing biological forms at the adult stage [32]. In this article, we analyzed *Obp1* intron I at the Ambouli 1 site in order to study the applicability of this method for differentiating biological forms. Surprisingly, molecular analysis yielded opposite results regarding the distribution of the type and mysorensis forms. Morphological analysis showed a large majority of the mysorensis form at this same collection site, while *Obp1* sequencing showed less than 1% of the latter. In support of the results presented here, a study conducted on samples from Sri Lanka had previously described a discrepancy between *Obp1* and morphological classifications of eggs [33]. In addition, a study conducted on *An. stephensi* collected in Nangarhar (eastern Afghanistan) identified mainly mysorensis and intermediate forms among mosquitoes infected with *P. falciparum* [34]. In India, similar results were described by Singh et al., where the authors found overlapping pairwise differences between biological forms and between specimens of the same form, concluding that the *Obp1* intron cannot be used as a biological marker to differentiate between them [35]. According to our results and those previously published in the literature, *Obp1* is not a suitable molecular tool for distinguishing between the three biological forms of *An. stephensi*.

Our study has several limitations: i) only a few eggs (326) from six sites were used for morphological analyses to assess the presence of different biological forms of *An. stephensi* in the city of Djibouti. (ii) No rural areas were included in this study. (iii) The relationship between polymorphism in the *Obp1* intron I region and the number of ridges on the eggs was not estimated on the same specimen, even though they were collected at the same time from the same breeding site. (iv) It was currently impossible to establish a link between the biological forms of *An. stephensi* and the malaria incidence rate at the selected sites, as this study was conducted in April 2023, whereas the malaria incidence data pertain to the year 2022. It would be interesting to know which biological form is responsible for malaria transmission, but this poses a significant challenge, as malaria infection rates are measured in adult female *Anopheles* mosquitoes, and biological forms must be determined based on the number of egg batches in their offspring.

## Conclusions

Our study is the first to describe the different biological forms of *An. stephensi* since its arrival in Djibouti in 2012 and its spread throughout East Africa. According to morphological and molecular analysis, *Obp1* is still not a suitable molecular tool for distinguishing between the three biological forms of *An. stephensi* which are sympatric in the breeding sites of urban and semi-urban areas of the city of Djibouti. The mysorensis form of *An. stephensi* was surprisingly found mainly in breeding sites located in an urban area with a high incidence of malaria, even though this form is described as a rural and ineffective vector of malaria. Further studies are needed to find a molecular tool that can distinguish between these biological forms of *An. stephensi* and identify the one responsible for malaria transmission in the Horn of Africa.

## Supporting information

S1 File Classification of the biological forms of *Anopheles stephensi* eggs based on the number of ridges. (XLSX)
